# Day occupation is associated with psychopathology for adolescents and young adults with Down syndrome

**DOI:** 10.1186/s12888-014-0266-z

**Published:** 2014-10-03

**Authors:** Kitty-Rose Foley, Peter Jacoby, Stewart Einfeld, Sonya Girdler, Jenny Bourke, Vivienne Riches, Helen Leonard

**Affiliations:** Telethon Kids Institute, University of Western Australia, Perth, Australia; School of Exercise and Health Sciences, Edith Cowan University, Perth, Australia; Department of Developmental Disability Neuropsychiatry, School of Psychiatry, The University of New South Wales, Sydney, Australia; Faculty of Health Sciences, University of Sydney, Sydney, Australia; Brain and Mind Research Institute, University of Sydney, Sydney, Australia; School of Occupational Therapy and Social Work, Centre for Research into Disability and Society, Curtin Health Innovation Research Institute, Curtin University, Perth, Western Australia Australia; Centre for Disability Studies, The University of Sydney, Sydney, Australia

**Keywords:** Intellectual disability, Down syndrome, Participation, Employment, Psychopathology, Behaviour

## Abstract

**Background:**

Young adults with Down syndrome experience increased rates of emotional and behavioural problems compared with the general population. Most adolescents with Down syndrome living in Western Australia participate in sheltered employment as their main day occupation. Relationship between day occupation and changes in behaviour has not been examined. Therefore, the aim of this research was to explore any relationship between post school day occupations and changes in the young person’s behaviour.

**Methods:**

The Down syndrome Needs Opinion Wishes database was used for case ascertainment of young adults aged 15 to 32 years with Down syndrome. Families of 118 young people in this population-based database completed questionnaires in 2004, 2009 and 2011. The questionnaires addressed both young person characteristics such as age, gender, presence of impairments, behaviour, functioning in activities of daily living, and family characteristics such as income and family functioning. Post-school day occupations in which the young people were participating included open and sheltered employment, training and day recreation programs. Change in behaviour of young adults who remained in the same post-school day occupation from 2009 to 2011 (n = 103) were examined in a linear regression model adjusting for confounding variables including age, gender, prior functioning and behaviour in 2004 and family income.

**Results:**

In comparison to those young adults attending open employment from 2009 to 2011, those attending day recreation programs were reported to experience worsening in behaviour both in the unadjusted (effect size −0.14, 95% CI −0.24, −0.05) and adjusted models (effect size −0.15, 95% CI −0.29, −0.01).

**Conclusions:**

We found that the behaviour of those participating in open employment improved compared to those attending other day occupations. Further examination of the direction of this association is required.

## Background

People with intellectual disabilities are at a higher risk of experiencing behavioural, emotional and psychiatric problems than the general population [1-3]. In an Australian study, approximately 40 percent of young people with intellectual disability aged 4 to 18 years were found to have severe emotional and behavioural disorders with a subsequent longitudinal study finding that psychopathology persisted over time [1,4]. People with Down syndrome have been reported to experience fewer behavioural and emotional disturbances than others with intellectual disability [5,6], yet still more than the general population [7]. Examination of age-related changes in behaviour of children and young people with Down syndrome revealed that externalising behaviours (dominant, opposing, impulsive) were more common in five to ten year olds and internalising behaviours (lacking in self-confidence/shy and insecure) more common in adolescents and adults (10 to 30 years) [8].

Behaviour problems have been found to be associated with poorer outcomes for young people with intellectual disability and to have a negative impact on social participation. Those in whom more behaviour problems have been reported were more likely to have activity limitations in communication, self-care and community skills [9-11]. They were also more likely to have difficulties forming and maintaining friendships and to spend fewer hours in education each month [12,13]. Moreover, poorer family outcomes have been reported for the families of those who have more behavioural problems. These include poorer family quality of life, family functioning and poorer maternal mental health [14-19].

According to social learning theory behaviour is learned through modelling, observing and imitating others [20]. One such place where this modelling, observing and imitating can occur is a person’s social environment within the workplace. Young people with intellectual disability who participate in different day occupations have varied opportunities to model, observe and imitate behaviours from peers [20]. Theorists have highlighted how changes in life-course, such as transitions, can impact on behaviour [21]. They discuss how relationships with peers and parents and participation in activities such as post-school day occupations can positively or negatively influence behaviour. The different day occupations in which young people with intellectual disability participate provide varied social environments and opportunities for modelling of behaviour, participating in activities and forming relationships with peers. These factors all have the potential to positively or adversely influence change in behaviour for young people with Down syndrome.

Post school day occupations for young people with intellectual disability in Australia include the following; open employment, i.e. work in a mainstream setting often with support; training, i.e. further education such as Technical and Further Education (TAFE); sheltered employment, i.e. work in a segregated setting for people with disabilities currently referred to as ‘Australian Disability Enterprises’ in Australia; Alternatives to Employment (ATE), i.e. a day recreation program specifically designed for people with disabilities who are unable to participate in employment or further training or; remaining at home with family or peers [10]. According to the Australian Institute of Health and Welfare in 2011 people with intellectual disabilities constitute 30% of the users of disability support services in Australia with 76% of those being under the age of 45 years [22]. They are able to access one of two government employment services 1) open employment services to access paid employment in the open labour market or 2) ‘supported employment’ services to access sheltered employment. Of all those who access the open employment services only 12% had an intellectual disability compared to 69% of those accessing the ‘supported employment’ services. Expenditure on disability support services has increased since 2005–2006, specifically community support services (by 80%) and employment support services (by 47%). Over the past ten years the participation of young people with intellectual disability in sheltered employment has increased by 25%. Participation in state government funded community access non-work programs such as ‘Alternatives to Employment’ (ATE) has also increased by 18%. However, the number of young people with intellectual disability participating in open employment has remained stagnant over this same time period [23], regardless of the reported 47% increase of expenditure on employment services since 2005 [22].

Identifying behaviour management strategies to reduce stress and enhance well-being for young people with Down syndrome has been highlighted as an important focus for research [2,24,25]. We know that the social environment can influence the behaviour of typically developing people [20], suggesting that the behaviour of young people with Down syndrome may also be influenced by different social environments, such as different day occupations (e.g. sheltered employment versus open employment). Therefore, the aim of our research was to explore the relationship between post school day occupations and young person’s change in behaviour.

The International Classification of Functioning, Disability and Health (ICF) is an internationally recognised framework for classifying health conditions, health related states and health outcome measurement [26]. Its usefulness for research in the field of intellectual disability has been well recognized [10,27-30]. Investigating complex experiences such as the relationship between behaviour and participation in day occupation taking into account the influence of environmental factors can present challenges. On this account, we have used the ICF to frame this research in order to examine these complex associations in a structured manner.

## Methods

The Down syndrome “Needs Opinions Wishes” database is a population-based source of young people with Down syndrome residing in Western Australia. This study focused on young people, ascertained from this database, aged 15 to 32 years in 2009, whose parents completed questionnaires at two time points: 2009 and 2011 (response fractions were 89%, 93%, respectively). Only those young adults who were post school in 2009 (n = 164) and/or 2011 (n = 180) were included in this study as we were interested in the relationship between post-school occupations and behaviour. A previous questionnaire in 2004 was used to obtain prior behaviour scores which were used in analysis to adjust for previous behaviour levels. There were 118 families whose sons and daughters were post school in 2009 and 2011 and who returned the questionnaire at all three time points. This number was further restricted to those young people who remained in the same day occupation from 2009 to 2011 (n = 103) to explore if there was a relationship between change in behavior and post-school day occupations.

**Figure 1:**
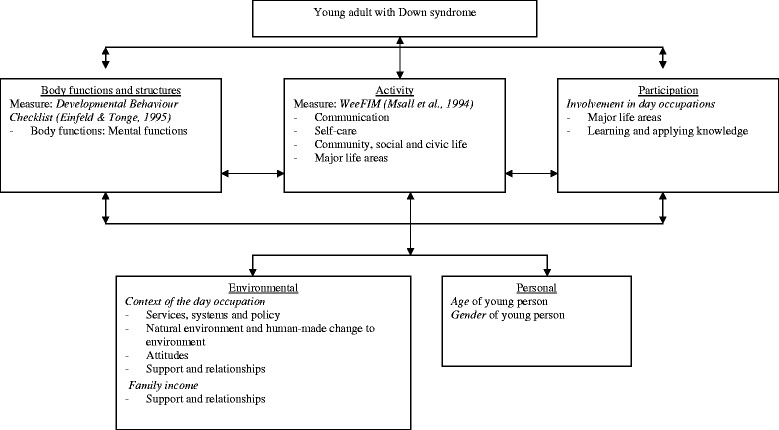
**Methodology model as per ICF framework: Second level classifications which are assessed within the ICF components.**

Data were collected in the form of questionnaires containing two parts. Part one pertained to the young person’s characteristics including age, gender, behavioural problems and functioning in activities of daily living (ADLs) and part two asked about family characteristics. The measures which were included in the questionnaire and are relevant for this study are classified within the components of the ICF. The relationships between the measures and the specific codes of the ICF for each component are shown in Figure 1.

### Body functions and structures

Behavioural and emotional problems were measured using the Developmental Behaviour Checklist (DBC), the 96-item child version in 2004 [31] and the 107-item adult version in 2009 and 2011 [32]. The DBC was specifically developed for people with developmental and intellectual disabilities with each behavioural response being scored 0 (not true as far as you know), 1 (somewhat or sometimes true) or 2 (very true or often true). The DBC-C has proven convergent validity, high inter-rater reliability between teachers and parents, high test-retest reliability and sensitivity to change [31]. The DBC-A has been found to have acceptable test-retest and inter-rater reliability and convergent ability has been demonstrated with two measures of behavioural disturbances of adults with intellectual disability [33]. The DBC-A and DBC-C were scored in three ways for this study which enables them to be comparable [34]; 1) Mean Item Score (MIS) reflecting the overall behaviour problems 2) Proportion of Items Checked (PIC) which is the proportion of items checked a one or a two and measures range of problem behaviours exhibited, 3) the Intensity Index (II) which is the proportion of items checked 2, out of all the items checked 1 or 2 which measures the severity of the problem behaviours.

### Activity

In the 2004 questionnaires, the Functional Independence Measure (WeeFIM), modified for questionnaire use as previously used [19,35], was included to measure functioning in activities of daily living [36]. The original WeeFIM has good inter-rater reliability and concurrent validity with the Pediatric Evaluation of Disabilities Inventory in children with developmental disabilities [37].

### Participation and environment factors

Day occupation is one aspect of the participation component of the ICF, identifying involvement in the different occupations. It is also associated with environment factors as the social and physical environment of the occupation could influence the young adults. Post school day occupations in which the young adults were participating in 2009 and 2011 were categorised according to open employment, training, sheltered employment or day recreation programs describing Alternatives to Employment (ATE) programs.

### Personal factors

Personal factors including date of birth and gender of the young adult with Down syndrome are ascertained from the database.

### Data analysis

Descriptive statistics including analysis of variance and chi-squared tests were used to describe the univariate relationship between independent variables and the outcome, change in problem behaviours from 2009 to 2011, for those who remained in the same day occupation from 2009–2011. Descriptive statistics were also used to describe the problem behaviour scores at each time point across the different post-school day occupations.

A linear regression model with change in behaviour from 2009 to 2011 as the outcome was used in the final model allowing adjustments for confounding variables. These included; age, gender, family income, previous functioning in activities of daily living and previous problem behaviour score. Unadjusted and adjusted models were reported separately. STATA 11 was used for these analysis [38].

## Results

### Participant characteristics

Of the young adults who were post school in 2009 (n = 164) and/or 2011 (n = 180), 143 (87%) had returned questionnaires at both time points. Of these 143, 118 individuals had also returned a questionnaire in 2004 when information on previous behaviours and functioning was utilised. For the 118, their ages ranged from 10 to 24 years in 2004 (mean 17.2 SD 4.3), 51 (43.2%) were female and 67 (56.8%) were male. It was noted that a number of individuals had left school in 2009 at younger than usual ages of 15–17 years. Out of the 118, there were 103 individuals who met the study criteria of being post-school, and remaining in the same day occupation from 2009 to 2011 and whose parents had also completed questionnaires in 2004.

### Relationship between post-school day occupations and change in behaviour problems

The day occupations of all young adults, who returned a questionnaire in 2009 and/or 2011, are shown in Table 1. Of the 103 young adults who remained in the same day occupation from 2009 to 2011, those attending day recreation programs were reported as having the poorest behaviour in 2009 in terms of range (PIC) (mean 0.30 SD 0.16), intensity (II) (mean 0.38 SD 0.21) and overall score (MIS)(mean 0.41 SD 0.25). From 2009 to 2011, the range (PIC (t(21) = −2.49, p = 0.02)) and overall score (MIS (t(21) = −1.98, p = 0.06) of behaviour problems of young adults participating in day recreation programs increased but the intensity (II (t(21) = 0.39, p = 0.70)) remained relatively stable (Table 2). In 2011, 45.5% (n = 10) of the young adults attending day recreation programs reported MIS scores beyond the cut-off point for psychiatric caseness, meaning a full psychiatric assessment is recommended [32].

**Table 1:** **Day occupations of all young adults who were post-school and returned questionnaires in 2009 and/or 2011**

**Day occupation**	**2009 (%)**	**2011 (%)**	**Remained**
Open employment	42 (25.6)	40 (22.2)	27 (26.2)
Training	17 (10.4)	23 (12.8)	8 (7.8)
Sheltered employment	64 (39.0)	75 (41.7)	46 (44.7)
Day recreation programs	41 (25.0)	38 (21.1)	22 (21.4)
Remained at home	0 (0)	4 (2.2)	-
Total	164 (100)	180 (100)	103 (100)

**Table 2:** **Mean scores of problem behaviour scores in 2009 and 2011**

		**Problem behaviour scores**								
		**Proportion items checked (range of behaviour problems)**			**Intensity index (severity of behaviour problems)**			**Mean item score**		
**Day occupations**	**Freq**	**2009 M, SD**	**2011 M, SD**	**p-value**	**2009 M, SD**	**2011 M, SD**	**p-value**	**2009 M, SD**	**2011 M, SD**	**p-value**
Open	27	0.14, 0.11	0.11, 0.10	0.049	0.15, 0.16	0.10, 0.22	0.317	0.16, 0.13	0.11, 0.10	0.016
Training	8	0.19, 0.15	0.22, 0.05	0.446	0.12, 0.15	0.15, 0.13	0.173	0.22, 0.21	0.25, 0.18	0.574
Sheltered	46	0.20, 0.15	0.17, 0.13	0.081	0.21, 0.20	0.19, 0.19	0.535	0.25,0.19	0.21, 0.17	0.114
Day recreation programs	22	0.30, 0.16	0.36, 0.12	0.021	0.38, 0.21	0.38, 0.24	0.982	0.41,0.25	0.50,0.35	0.061

Note: Higher behaviour score refers to poorer behaviour from 2009 to 2011.

Note: Cut-off score for psychiatric caseness MIS = 0.48.

Young adults attending open employment in 2009 were reported as having the fewest behavioural problems in terms of range (PIC (mean 0.14, SD 0.11)) and overall score (MIS (mean 0.16 SD 0.13)) and those attending training had the lowest intensity (II (mean 0.12 SD 0.15)) of behavioural problem. The range (PIC(t(26) = 2.07, p = 0.049)) and overall (MIS (t(26) = 2.58, p = 0.016)) scores for those attending open employment decreased significantly from 2009 to 2011. The range (PIC (t(45) = 1.78, p = 0.08)), intensity (II (t(44) = 0.87, p = 0.54)) and overall score (MIS (t(45) = 1.61, p = 0.11)) for behaviour problems in those attending sheltered employment showed a similar but not significant trend to decrease from 2009 to 2011 (Table 2).

### Adjusted model

Change in behaviour from 2009 to 2011 was converted to a change score for the regression model, where a positive number referred to an improvement in behaviour (Table 3). Confounding variables which were adjusted for included age in 2004, gender, family income, functioning in activities of daily living (ADL) in 2004 and problem behaviours (DBC continuous score) in 2004. The effect size in the table reflects a per point change in overall behaviour MIS compared to the change in behaviour of those attending open employment. In comparison to those young adults attending open employment from 2009 to 2011, those attending day recreation programs experienced significant worsening in behaviour both in the unadjusted (effect size −0.14, 95% CI −0.24, −0.05) and adjusted models (effect size −0.15, 95% CI −0.29, −0.01)(Table 3).

**Table 3:** **Linear regression models of behaviour change scores from 2009 to 2011 by day occupation**

		**Unadjusted (n = 103)**			**Adjusted model (n = 69)**		
		**Effect size** ^**†**^	**95% CI**	**P value**	**Effect size** ^**†**^	**95% CI**	**P Value**
Mean item scores	Open employment	Baseline			Baseline		
	Training	−0.08	−0.21, 0.05	0.217	−0.10	−0.23, 0.04	0.152
	Sheltered employment	−0.01	−0.09, 0.06	0.728	−0.01	−0.10, 0.09	0.894
	Day recreation programs	−0.14	−0.24, −0.05	0.002	−0.15	−0.29, −0.01	0.034

Note: Variables in adjusted model: age, gender, family income (imputed variable), functioning in 2004, behaviour in 2004.

Note: Positive behaviour score refers to an improvement in behaviour from 2009 to 2011.

^†^Effect size is the estimated difference in the behaviour change score between each group and the reference group (open employment).

We examined the change in behaviour of the young adults who were in open employment or day recreation programs at the 2009 time point but were not in the same occupation in 2011. We were interested to compare the behaviour change patterns of those young adults who did not remain in the same day occupation with those who did. We found that the patterns were similar for those who remained in open employment (mean change 0.05, SD 0.10) and those that changed from open employment to a different day occupation (mean change −0.02 SD 0.17)(p-value = 0.14). However, the behavior of those who remained in day recreation programs deteriorated (mean change −0.09 SD 0.22) compared with those that moved out of day recreation programs into a different day occupation (mean change 0.05 SD 0.14)(p = 0.05).

## Discussion

Adolescents and young adults with Down syndrome attending open employment for two consecutive years were found to experience a decline in behaviour problems in terms of range, intensity and overall problems, after adjusting for known confounding variables. Those attending sheltered employment for two years also experienced a decline in problem behaviours in range, intensity and overall behaviour problems, but this was less marked than for those in open employment. Young adults who were attending day recreation programs for two years experienced an increase in range, intensity and overall behaviour problems. At the second time point almost half of these young adults’ behaviour problems were reported to be beyond the clinical cut-off score for psychiatric caseness [32].

A considerable strength of this paper is the use of the DBC to measure emotional and behavioural problems at three time points. The use of the child version and the adult version ensures that the questionnaire remains applicable and valid. Scoring the range and intensity of emotional and behavioural problems adds a particularly clinically relevant interpretation of the data which could not be ascertained from only scoring the overall total [34]. It allows us to recognize the type of behaviour changes which then provides more detailed information to guide development of intervention. A limitation of this study relates to the fact that those young people who move out of open employment could do so because of deteriorating behaviour, which could contribute to the improved behaviour seen in the group who remain. However, when we investigated this we found that there was no difference in the changes in behaviour of those that remained and those that left open employment. However, we did find that for those who left the day recreation programs behaviour improved significantly in comparison to those who remained in day recreation programs. We cannot definitely state whether these young people’s behaviour improved because they left the day recreation programs or they left because their behaviour improved. Another potential limitation of this study is that the data are parent report. Research in the general population has indicated that there may be discordance between parent and young person reporting of emotional and behavioural problems, specifically in regards to internalizing behaviours and when the parent experiences psychopathological issues [39-41]. However, challenges gaining self-report data from young people with intellectual disability have been acknowledged and the need for appropriate and psychometrically rigorous instruments for young people with intellectual disability to report their own emotions and behaviours has been highlighted [42,43]. It is important to investigate the potential influence that participation in open employment could have on behavioural and emotional problems of young adults with Down syndrome. Although, we cannot definitively confirm any causal relationship between day occupation and change in behaviour over time, our study would suggest that further scrutiny of this association is needed. More detailed information on the length of time spent within particular jobs may also help elucidate the factors affecting behaviour.

We found that participation in open employment was associated with an improvement in behaviour over time compared to other day occupations. This association could be attributed to many different factors such as the modeling of positive behaviours from typically developing peers or the satisfaction of participation in a meaningful, mainstream occupation. The idea that the behavior of young people who are attending open employment improves as a result of modeling, observing and imitating the behavior of their typically developing peers is supported by the theory of social learning [20]. Research has shown that young people with intellectual disability who participate in open employment experience greater perceptions of job clarity and are provided more opportunities for socialization than those participating in day recreation programs or sheltered employment [44,45]. Previous research has already shown that there are positive associations between participation in open employment and social and activity related outcomes and that young people with intellectual disability have a desire to have the opportunity to participate in the open labour market [46,47]. Despite this evidence and the significant increase in expenditure on employment services for people with disabilities in Australia, there has not been a change in the number of people with intellectual disability participating in open employment in Australia over the past ten years [22,23].

In relation to sheltered employment, our results showed a trend towards improving behaviour over the two year time period. Martorell, Gutierrez-Recacha, Pereda and Ayuso-Mateos (2008) examined behaviour of young people involved in sheltered employment and day recreation services through a cross-sectional study and concluded that those participating in sheltered employment reported less problem behaviours compared to those attending day recreation services. However, people who were attending open employment were not included in their study and the authors proposed that behaviour problems “preclude a good functioning, hence causing a worse work outcome” [48]. We suggest that the direction of this relationship has not been proven and perhaps young people who have more problem behaviours could decrease problem behaviours through participation in open employment. In a further adjusted analysis Martorell and colleagues showed that the influence of behaviour was ameliorated by the inclusion of a variable describing self-determination. Self-determination has been reported to occur with more normalized, community-based environments for people with intellectual disability, such as an open employment context [49,50].

In our study the young adults who were attending day recreation programs for two consecutive years showed a concerning increase in range of problem behaviours and overall problem behaviours. This could be attributed to lack of choice-making opportunities, isolation and segregation from the community and lack of meaningful and challenging activities within the day recreation programs. These young adults showed a significant increase in range of problem behaviours but not intensity. This also suggests that the young adults who were attending day recreation programs may have modelled undesirable behaviours from their peers in the day recreation programs environment which would increase the range of problems they exhibit and not alter the intensity. Additionally, almost half of those young adults attending day recreation programs for two years had reported problem behaviour scores beyond the clinical cut-off for a psychiatric case. The developers of the DBC recommend a comprehensive psychiatric assessment is recommended for those young adults who scored beyond the cut-off score [32]. The stated aims of day recreation programs include support outcomes related to social participation, increasing independence, lifelong learning and enhanced support networks [51]. The findings from this study must be interpreted in the context of the limitations of the study, yet definitely warrant further examination of whether the aims of the day recreation programs are currently being met.

Framing this research within the ICF allows for investigation of the ICF components which have an association with change in behaviour for young people with intellectual disability. This research has highlighted the potential for environmental factors (i.e. context of the day occupations) to modify behavioural disturbances in young adults with Down syndrome. We cannot confirm the direction of the relationship between change in behaviour and day occupation. However our findings do raise specific questions about the potential mechanisms underlying these. We also found a trend towards decreasing problem behaviours for young adults who were attending sheltered employment compared to other day occupations. The main difference between a sheltered employment environment and a day recreation program environment is participation in an organized task and adherence to routines and clearly defined rules for safety, dress and behaviour. The increase in problem behaviours in those young adults participating in day recreation programs compared to those participating in sheltered employment suggests that the activity of undertaking specific tasks in the sheltered employment environment could be playing a role in decreasing problem behaviours for those young people. The sheltered employment environment could also create an opportunity for steady friendships which could have a positive influence on behaviour. These points highlight the relationship between the participation component of the ICF and the impairments of body functions and structures component. They also provide valuable information about the importance of environmental factors and participation when considering the psychopathology of young people with Down syndrome.

## Conclusion

The problem of psychopathology has been reported as both substantial and persistent for young people with intellectual disability and the need for effective mental health interventions is paramount [4]. This study has identified an association between improvements in behavior problems and participation in open employment for young people with Down syndrome, while adjusting for known confounding variables. This finding provides information which could be helpful when developing mental health interventions for young people with Down syndrome. This study is one of the first, to the authors’ knowledge, to investigate the relationship between behavioural change and specific post-school day occupations. The longitudinal nature of the study adds strength as well as the fact that case ascertainment occurred from a population-based database. Future research should focus on identifying the specific mechanisms within an open employment setting which could positively influence behaviour change.
